# Health Professionals' Knowledge and Perceived Clinical Skill Toward Emergency Preparedness and Its Associated Factors in North Showa, Ethiopia, 2024

**DOI:** 10.1002/hsr2.71854

**Published:** 2026-02-25

**Authors:** Melese Wagaye Zergaw, Makda Abate Belew, Ayele Tilahun, Hailegiorgis Geleta Abocherugn, Sisay Tulu Ruksi, Dejene Hailu Beyene

**Affiliations:** ^1^ Department of Nursing, School of Nursing and Midwifery, Asrat Woldeyes Health Science Campus Debre Berhan University Debre Berhan Ethiopia; ^2^ Department of Nursing, College of Health Sciences Salale University Fiche Ethiopia; ^3^ Department of Midwifery, College of Health Sciences Mizan Tepi University Tepi Ethiopia

**Keywords:** disaster, Ethiopia, experience, health professionals, knowledge, training needs

## Abstract

**Background:**

Emergency and public health problems are unavoidable and can strike at any time. In a globalized world where crises are getting more common, more devastating, and have a substantial influence on society's health and life, the quality of healthcare services is becoming increasingly important. Such an emergency causes a double burden of impact, especially for African states, due to their low economies and burden of ill health.

**Objectives:**

To assess health professionals' knowledge and perceived clinical skill toward emergency preparedness and its associated factors in North Showa, Ethiopia, 2024.

**Methods:**

An institution‐based cross‐sectional study design was employed from December 1 to January 30, 2024. Data was collected using a pre‐tested and structured questionnaire from 424 study participants. Statistical analysis was performed using SPSS version 25 software. On bivariable logistic regression analysis, variables with *p*‐value < 0.25 were transferred to multivariable logistic regression analysis and variables with *p*‐value < 0.05 were considered as statistically significant. The result was summarized using tables, graphs, and charts for different variables.

**Results:**

A total of 424 participants were enrolled in the study, with a response rate of 413 (97.4%). Among 413 study participants, only 57.6% participants had adequate knowledge of emergency preparedness. Midwifery (AOR = 0.097; 95% CI 0.033–0.284), laboratory (AOR = 0.244; 95% CL 0.073–0.812), pharmacy (AOR = 0.078; 95% CI 0.021–0.288), disaster volunteer (AOR = 0.542; 95% CI 0.318–0.924), having 1–5 years of experience (AOR = 2.541; 95% CL 1.479–4.365), and > 30 years of age (AOR = 3.694; 95% CI 2.026–6.733) were significantly associated with adequate knowledge toward emergency preparedness.

**Conclusion and Recommendations:**

About 57.6% of health professionals working in North Showa, Oromia, Ethiopia had adequate knowledge of emergency preparedness. Midwifery, laboratory, pharmacy, disaster volunteering, 1–5 years of experience and > 30 years of age were significantly associated factors. Therefore, training regarding emergency preparedness should be given to all health professionals, including midwifery, medical laboratory and pharmacy, to increase their knowledge level. The local health department also recommended working on creating many disaster volunteer health professionals to solve the problem.

**Abbreviations**
AOR: adjusted odds ratio
CI: confidence interval
DPPA: disaster prevention and preparedness agency
IESO: integrated emergency surgery officer
IRB: Institutional Review Board
MCIs: multiple casualty incidents
NGOs: non government organizations
PAHO: Pan American Health Organization's
SPSS: Statistical Package for the Social Sciences
WHO: World Health Organization's

## 1 Introduction

### 1.1 Background

In today's increasingly interconnected world, no community or country can be protected from public emergencies and disasters [1]. Emergency and public health problems are unavoidable and can strike at any time. A well‐established readiness program is required for healthcare systems, particularly hospitals, to respond effectively to catastrophes [2, 3]. These problems are exacerbated by factors such as population shifts from rural to urban regions, environmental degradation, and rapid changes in land use. These socioeconomic conditions, together with the country's proclivity for natural disasters such as earthquakes, floods, and landslides, among others, exacerbated by human activities and shifting climate conditions, corroborate a long‐term trend [4].

People all across the world are at risk of a variety of health‐related and natural‐disaster‐related hazards. Among them are infectious disease outbreaks, natural disasters, conflicts, contaminated food and water, chemical and radiation incidents, building collapses, transportation incidents, a lack of water and power, air pollution, antimicrobial resistance, climate change effects, and other sources of risk [5, 6, 7]. Although natural disasters cannot be stopped or controlled, citizens must be prepared at all levels, including individuals, families, healthcare workers, and community groups, to launch a successful response [8].

Globally, around 1.6 million individuals have died as a result of catastrophes, which cause approximately 65,000 deaths every year. Such an emergency causes a double burden of impact, especially for African states, due to their low economies and burden of ill health. African nations also have an increasing impact on communicable diseases. This creates for Ethiopia, and the whole African continent, a vicious cycle of disaster and emergency events (UNISDR, 2015). Actions and policies must be implemented to increase society's resilience to natural disasters and ensure that vulnerability to hazards does not rise as a result of development to meet the goal of minimizing the risks of natural hazards to social, environmental, and economic assets. Preparing for a disaster or emergency is crucial, and it should be a top priority for everyone to think about. Individuals or experts in every field must be knowledgeable and equipped to plan for and respond to emergencies.

### 1.2 Statement of Problems

In 2015, 574 verified catastrophes were caused by earthquakes, floods, landslides, and heat waves, killing roughly 32,550 people, injuring over 108 million people, and generating $70.3 billion in damage [9]. In 2019, 143 significant floods struck, accounting for more than half of the year's total catastrophic disasters, killing 5076 people (43.4%) and affecting 29,634,800 people (32.8%). There were 61 major storm disasters, accounting for over 21% of all major disasters, killing 2519 people, hurting 31.29 million people, and generating $57.9 billion in direct economic losses (48%). Every year, more than 1.2 million people die, and 50 million are injured on the world's roads [9, 10].

Several Asian countries (India, Nepal, Bangladesh, and Myanmar) have all suffered from severe flooding, with thousands of people dying from monsoon flooding, while the United States suffered economic losses of more than $20 billion from flooding in 2019 [9]. Cyclones and storms, floods, landslides, severe temperatures, wildfires, and droughts are the most common hydro‐meteorological and climatological disasters in Sub‐Saharan Africa. Drought and flooding are responsible for 80% of natural disaster deaths and 70% of economic losses in Sub‐Saharan Africa [11].

Ethiopia is one of the 20 countries identified as having the highest vulnerability to natural hazards and low economic resilience. Ethiopia has one of the highest rates of road traffic fatalities in the world, with 25.3 fatalities per 100,000 people [12, 13, 14]. Ethiopia and Kenya, with almost 61 million and 48 million people, respectively, accounted for 30% of the overall number of individuals affected [15]. Furthermore, flooding caused by river bank overflow in South Omo claimed the lives of 360 people and harmed the productivity and livelihoods of over 20,000 people in the Dasanach and Ngangatom districts [16].

The occurrence of multiple casualty incidents (MCIs), such as swine flu pandemics, has transformed healthcare workers' perception toward disaster preparedness. Countries were encouraged to respond to the World Health Organization's (WHO) and Pan American Health Organization's (PAHO) request to take significant steps to ensure safe health care facilities during an emergency. This response must include evaluating hospital safety, training and protecting health personnel for emergencies, planning, and emergency response strategy, designing and building long‐lasting hospitals, implementing national programs and policies, and safeguarding medical and ordinary equipment, materials, and supplies [17]; (WHO, 2013).

Despite improved public awareness toward the MCIs, threats, and various guidelines recommended for hospital disaster plans by several agencies, the emphasis on preparing the healthcare workforce for such disasters is inadequate [4]. Physicians and nurses comprise the highest percentage of the health and medical workforce. They must understand the national disaster management cycle. Without their integration at every phase, communities and clients lose a critical part of the prevention network (Shabbir et al. 2017) [4, 18, 19].

Emergency preparedness and response mechanisms, such as those for outbreak alert and response and mass casualty management, must be tested and evaluated regularly at each level of the health system. Countries and communities should seize post‐event recovery opportunities to strengthen capacities and reduce the risk of future emergencies through effective planning and long‐term implementation of rehabilitation and reconstruction measures. Thus, the present study will be aimed to examine health professionals' knowledge and perceived clinical skill toward disaster preparedness and its associated factors in North Showa zone, Oromia, Ethiopia.

### 1.3 Significance of the Study

Reducing emergency is one of the big challenges that needs up‐to‐date information on emergencies and disasters for a better understanding of the problem and serves for the development of an effective preventive strategy.

The findings of this study will assist the researcher, health professionals, different NGOs, stakeholders, and community health organizers to comprehend emergency status in the study area and help them to prioritize their plan and to develop action plans and interventions for emergency management accordingly. Additionally, this study informs priority areas of health managers of the North Showa and is helpful to mobilize and plan government mental health initiatives aimed at screening and early intervention.

## 2 Objectives of the Study

### 2.1 General Objective

To assess health professionals' knowledge and perceived clinical skill toward emergency preparedness and its associated factors in North Showa, Ethiopia, 2024.

### 2.2 Specific Objectives

- To determine the level of health professionals' knowledge of emergency preparedness in North Showa.
- To identify factors associated with health professionals' knowledge of emergency preparedness in North Showa.

## 3 Methods and Materials

### 3.1 Study Area

The study was conducted in the North Showa Zone of Oromia Regional State. Fiche is the capital city of the northern Showa zone, which is located at a distance of 112 km from Addis Ababa in the northern direction. North Showa is surrounded by Amara regional state in the North and East, Addis Ababa special zone in the South and West Showa zone in the West. North Showa Zone has a total population of 1,639,587, where 820,595 were male and 818,992 were female. Eighty‐eight percent (1,447,330) of the North Showa's population lives in rural areas and 12% (192,105) live in urban areas. The North Showa Zone has two general hospitals and three district hospitals, 63 health centers, and 268 health posts. It has seven medium clinics, 54 lower clinics, one drug store, 25 drug vendors, and three rural drug vendors.

### 3.2 Study Design and Period

An institution‐based cross‐sectional study design was employed from December 1 to January 30, 2024.

### 3.3 Population

#### 3.3.1 Source Population

All health professionals working at North Showa zone health facilities were the source population.

#### 3.3.2 Study Population

All health professionals working at North Showa Zone health facilities and fulfilled inclusion criteria were the study population.

### 3.4 Eligibility Criteria

#### 3.4.1 Inclusion Criteria

Health professionals working in the selected health facility and available at the time of data collection were included.

#### 3.4.2 Exclusion Criteria

Participants who are unable to provide information for a variety of reasons, such as illness, were excluded as well.

### 3.5 Sample Size Determination

The sample was calculated using a single population proportion formula using proportional knowledge 50.8% (424) and practice 8.3% (129) on the emergency from a study done at Tikur Anbessa Specialized hospital [20] and considering 95% confidence interval and 5% marginal error with 10% non‐response rate, the total sample size was 424.

### 3.6 Sampling Technique and Procedure

**FIGURE 1:**
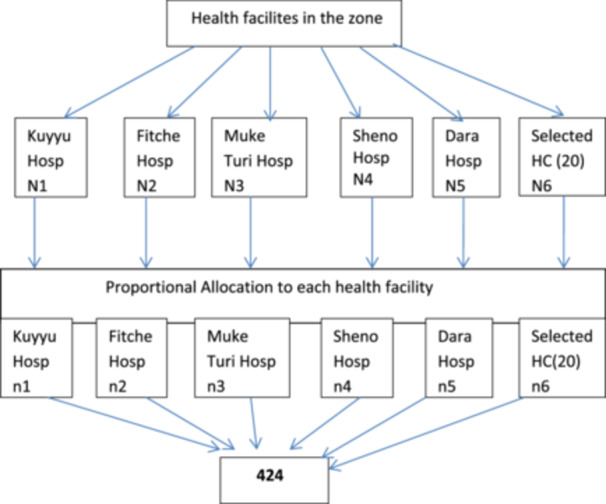
Schematic presentation of sampling procedures on health professionals' knowledge and perceived clinical skill toward emergency preparedness and its associated factors in North Showa, Ethiopia.

The sample was proportionally allocated to each public hospital (Fitche, Kuyu, Muketuri, Sheno, and Gundomeskel hospitals) and 20 public health centers based on a proportional allocation formula. As a sampling frame, a list of all health professionals working at the hospital and selected health centers were used. Finally, a simple random sampling technique using a lottery method was used to select the participants by using their salary payroll lists from the human resource office of each hospital as a sampling frame (Figure 1).

### 3.7 Data Collection Tools and Producers

The data was collected using a self‐administered questionnaire after it was adapted from previous studies [20, 21, 22, 23] and modified accordingly, then developed in English and translated into the local language Afaan Oromo, and then translated back to English to check the consistency. The questionnaire included socio‐economic and demographic characteristics of respondents, knowledge, and perceived clinical skills about the disaster. The questions particularly focused on common disasters, including drought, floods, epidemic diseases, fire, and traffic accidents. Seven B.Sc. nursing will be recruited (five for data collection and two for supervision). Besides, they will be trained on the ethical principles of confidentiality and data management.

### 3.8 Data Quality Control

Quality of data was ensured during collection, coding, entry, and analysis. To increase the quality of data during data collection, three teams, namely data collectors, supervisors and investigators, will be assigned and properly followed for its completeness. A pre‐test will be conducted on 5% of the sample size at Cancho Hospital to check for consistency of the tool and modifications accordingly. Training will be given for 2 days for the data collectors and supervisors to prevent any confusion and have a common understanding of the tool.

### 3.9 Data Entry and Analysis

Data are checked after collection for completeness and consistency, then coded and entries will be made in computer software Epi Info version 7 statistical packages. After cleaning the data, the statistical analysis will be performed using SPSS version 23. Descriptive statistics will be computed. Summary values, such as frequency, percentage, mean, or median, will be used to describe the study findings. The results will be presented using frequency tables and graphs. The bivariate analysis will be employed to select candidate variables for multivariable logistic regression. Variables with *p* values of less than 0.25 will be entered into multivariable logistic regression. To control the effect of confounding factors, multivariable logistic regression will be conducted. *p*‐value < 0.05 will be considered as statistically significant for all the independent variables in the final model.

### 3.10 Operational Definitions

**Emergency**—An expected and difficult or dangerous situation, especially an accident which happens suddenly that requires quick action from health professionals.

**Preparedness**—The capacities and knowledge developed by governments, professional response organizations, communities and individuals to anticipate and respond effectively to the impact of likely, imminent or current hazard events or conditions.

**Adequate knowledge**—Refers to respondents who have scored more than or equal to 50% of knowledge questions [20].

**Inadequate knowledge**—Refers to respondents who have scored less than 50% of knowledge questions [20].

### 3.11 Expected Outcome

When this study is conducted, it is expected to answer the following points:

Health professionals' knowledge and clinical skills regarding emergency preparedness will be identified.

Factors affecting health professionals' emergency preparedness will be identified.

Based on the research findings, appropriate intervention will be designed to alleviate the problem in the study area.

## 4 Results

### 4.1 Socio‐Demographic Characteristics

Overall, 424 participants were included in this study with a response rate of 413 (97.4%). Of 413 study participants, 347 (84%) were males and 230 (55.7%) respondents were married, 151 (36.6%) were single, and 32 (7.7%) were divorced and separated. The mean age of the respondents was 28.44± (SD = 3.54) (Table 1).

**TABLE 1:** Socio‐demographic characteristics of the respondents in North Showa public hospitals, Oromia, Ethiopia 2024 (*n* = 413).

Variables		Frequency	Percent (%)
Sex	Male	347	84
	Female	66	16
Age	20–30	296	71.7
	≥ 30	117	28.3
Marital status	Married	230	55.7
	Single	151	36.6
	Other*	32	7.7
Educational status	Diploma	72	17.4
	Degree	336	84.1
	Other	5	1.2
Working institution	Hospital	396	95.9
	Health center	9	2.2
	Health office	8	1.9
Average monthly income	< 5000	113	24.7
	5000–10,000	285	69
	≥ 10,000	15	2.6
Profession	Nursing	240	58.1
	Midwifery	50	12.1
	Medical laboratory	27	6.5
	Medicine	40	9.7
	Pharmacy	27	6.5
	Other	29	7.0
Service year	< 1 year	105	25.4
	5–6 year	213	51.6
	≥ 6 year	95	23

*Note:* Other*; divorced/separated.

### 4.2 Knowledge of Respondents Toward Emergency Preparedness

**FIGURE 2:**
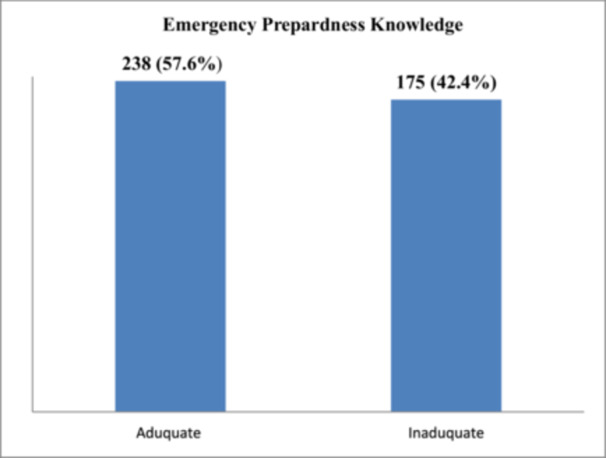
Knowledge of respondents toward emergency preparedness in North Showa, Oromia, Ethiopia 2024 (*n* = 413).

Among 413 study participants, 238 (57.6%) participants had adequate knowledge of emergency preparedness (Figure 2).

### 4.3 Institutional Related Factors

Out of the total, study participants, 213 (51.6%), 105 (25.4%), and 95 (23.0%) had 1–5, less than 1, and greater than 6 years of experience, respectively. Of 413 participants, 364 (88.1%) were working as technical staff and 49 (11.9%) were working as head of the organization and program coordinator. Out of the total participants 81 (19.6%) got training regarding emergency preparedness, whereas 332 (80.4%) did not get training toward emergency preparedness.

### 4.4 Occurrence of Common Disasters as Reported by Health Professionals

The participants were asked to rate the frequency of occurrence of common disasters in their locality. Transport accidents, disease epidemics, infestations, environmental pollution, fire, drought, flood, landslide, earthquake and explosion, in that order of importance, were mentioned as common disasters. Explosions and earthquakes were reported to be less frequent (Table 2).

**TABLE 2:** Occurrence of common disasters as reported by health professionals in North Showa, Oromia, Ethiopia, 2024 (*n* = 413).

		Frequency		
	Very frequent	Frequent	Less frequent	Not at all
Disaster category	No. (%)	No. (%)	No. (%)	No. (%)
Transport accidents	92 (22.3)	163 (39.5)	137 (33.2)	137 (33.2)
Disease epidemic	17 (4.1)	69 (16.7)	272 (65.9)	55 (13.3)
Infestations	25 (6.1)	79 (19.1)	189 (45.8)	120 (29.1)
Environmental pollution	83 (20.1)	146 (35.4)	150 (36.3)	34 (8.2)
Fire	26 (6.3)	51 (12.3)	253 (61.3)	83 (20.1)
Drought	16 (3.9)	47 (11.4)	251 (60.8)	99 (24.0)
Flood	8 (1.9)	22 (5.3)	172 (41.6)	211 (51.1)
Landslide	—	35 (8.5)	60 (14.5)	318 (77.0)
Earthquake	13 (3.1)	9 (2.2)	32 (7.7)	359 (86.9)
Explosion	21 (5.1)	12 (2.9)	93 (22.5)	287 (69.5)

### 4.5 Health Professionals' Needs for In‐Service Short Course Training on Emergency Preparedness

Two hundred nine (50.8%) of study participants have said that field epidemiology or ecosystem health is very important. Among these 209 participants, 126 (60.3%) had adequate knowledge of emergency preparedness. Among study participants, responding communication skills are very important, only 123 (55.2%) had adequate knowledge (Table 3).

**TABLE 3:** Health professionals' needs for in‐service short course training on emergency cross tab in North Showa, Oromia, Ethiopia 2024 (*n* = 413).

Variables		Adequate knowledge	(%)	Inadequate knowledge	(%)
Communication skill	Not important	5	55.6	4	44.4
	Little important	—	—	4	100
	Important	110	62.1	67	37.9
	Very important	123	55.2	100	44.8
Disaster management	Little important	—	—	8	100
	Important	83	68.6	38	31.4
	Very important	155	54.6	129	45.4
Resource mobilization & health economics	Little important	—	—	8	100
	Important	86	67.2	42	32.8
	Very important	152	54.9	125	45.1
Risk analysis	Not important	—	—	5	100
	Little important	8	34.8	15	65.2
	Important	72	53.7	62	46.3
	Very important	151	60.2	100	39.8
Leadership skills	Not important	—	—	4	100
	Little important	4	16.0	21	84.0
	Important	91	62.8	54	37.2
	Very important	143	59.8	96	40.2
Epidemiology and ecosystem health	Not important	8	61.5	5	38.5
	Little important	11	47.8	12	52.2
	Important	93	55.4	75	44.6
	Very important	126	60.3	83	39.7

### 4.6 Practice

Among 413 participants, only 27(6.5%) of participants said yes to having their ongoing training in their facilities and 167(40.4%) didn't face any disasters during their stay (Table 4).

**TABLE 4:** Frequency distribution of health professionals' practice score toward emergency preparedness in North Showa, Oromia, Ethiopia 2024 (*n* = 413).

Variables	Yes no. (%)	No no. (%)	I don't know no. (%)
Disaster drill done at your hospital?	77 (18.6)	246 (59.6)	90 (21.8)
Is there ongoing training?	27 (6.5)	340 (82.3)	46 (11.1)
Is the disaster plan periodically updated?	38 (9.2)	247 (59.8)	128 (31.0)
Have you ever been faced any disaster?	193 (46.7)	167 (40.4)	53 (12.8)
Have you ever been a worker for disaster management team?	55 (13.3)	283 (68.5)	75 (18.2)
Do you know about the latest Disaster in which your hospital involved?	158 (38.3)	178 (43.1)	77 (18.6)
Do you believe your practice for disaster preparedness is insufficient?	222 (53.8)	135 (32.7)	56 (13.6)

### 4.7 Factors Associated With Health Professionals' Self‐Rated Knowledge About Emergency Preparedness

Bivariate logistic regression analysis was performed to identify variables associated with knowledge of health professionals toward emergency preparedness at *p* value < 0.25 and variables with *p* value < 0.25 were enrolled in multivariable logistic regression. Five variables were associated with knowledge of a health professional toward emergency preparedness at *p*‐value < 0.25. Those variables were marital status, type of profession, disaster volunteering, age and service year. After adjustment for potential confounders, type of profession, disaster volunteering, age and service year were significantly associated with health professionals' knowledge of emergency preparedness.

At *p*‐value 0.000, about 9.7% of midwifery health professionals had adequate knowledge of emergency preparedness (AOR = 0.097; 95% CI 0.033–0.284), 2.4% of health professionals with medical laboratory specialty had adequate knowledge toward emergency preparedness at *p*‐value 0.021 with (AOR = 0.244; 95% CL 0.073–0.812), whereas 7.8% of pharmacy health professional participants had adequate knowledge toward emergency preparedness at *p*‐value 0.000 with (AOR = 0.078; 95% CI 0.021–0.288) than ISEO, Ophthalmic nurse, anesthesia, and radiography health professionals. Nearly 5.4% of participants with disaster volunteers had adequate knowledge of emergency preparedness at *p*‐value 0.024 with (AOR = 0.542; 95% CI 0.318–0.924) than non‐volunteer while participants who had 1–5 years of experience were nearly 2.5 times (AOR = 2.541; 95% CL 1.479–4.365) more likely to have adequate knowledge toward emergency preparedness at *p*‐value 0.001 than less than 5 years of experience. Respondents greater than 30 years of age were nearly 3.7 times more likely to have adequate knowledge of emergency preparedness at *p*‐value 0.000 (AOR = 3.694; 95% CI 2.026–6.733) than those greater than 20–30 years of age at *p*‐value 0.000 (Table 5).

**TABLE 5:** Factors associated with health professionals' knowledge about emergency preparedness North Showa, Oromia, Ethiopia, 2024 (*n* = 413).

Variables	Category	Knowledge		COR (95% CI)	AOR (95% CI)	*p*‐value
		Adequate	Inadequate			
Profession	Nursing	158	82	0.734 (0.312–1.729)	0.501 (0.201–1.249)	0.138
	Midwifery	17	33	0.196 (0.072–0.535)	0.097 (0.033–0.284)	**0.000**
	Laboratory	14	13	0.410 (0.135–1.245)	0.244 (0.073–0.812)	**0.021**
	Medicine	23	17	0.515 (0.184–1.440)	0.495 (0.166–1.481)	0.209
	Pharmacy	5	22	0.087 (0.024–0.307)	0.078 (0.021–0.288)	**0.000**
	Other*	21	8	1		
Disaster volunteering	Yes	168	142	0.558 (0.348–0.893)	0.542 (0.318–0.924)	**0.024**
	No	70	30	1		
Service year	< 1	49	56	1		
	1–5	137	76	2.060 (1.281–3.313)	2.541 (1.479–4.365)	**0.001**
	> 5	52	43	1.382 (0.792–2.412)	0.774 (0.392–1.528)	0.460
Age	20–30	154	142	1		
	> 30	84	33	2.347 (1.478–3.728)	3.694 (2.026–6.733)	**0.000**

*Note:* Other* IESO, ophthalmic nurse, anesthesia, and radiography; CI, confidence interval; AOR, adjusted odds ratio; COR, crude odds ratio. Bold values indicates significantly associated.

## 5 Discussion

### 5.1 Knowledge of Health Professionals Toward Emergency Preparedness

This cross‐sectional study was conducted with the aim of determining the knowledge of health professionals regarding emergency preparedness and associated factors. The knowledge of health professionals toward emergency preparedness was 57.6% (95% CI 53.3–62.4). This finding was consistent with the study findings reported in Coastal Areas of Indonesia [24].

However, the finding was lower than the study findings reported in Shanghai, China [25]. The discrepancy may be due to differences in sociodemographic status and availability of training access regarding emergency preparedness. Additionally, there may also be exposure to different types of disasters compared to Ethiopia.

The findings of this study were higher than the cross‐sectional study findings done at Tikur Anbessa specialized Hospital, Ethiopia [20]. The reason may be justified as our study was conducted at the Zonal level, which includes many hospitals, whereas study findings from Tikur Anbessa specialized Hospital were conducted in a single hospital. Additionally, there were 8 years of gap between those studies.

Regarding the training needs, only 19.6% of health professionals got training regarding emergency preparedness, whereas 80.4% did not get training for emergency preparedness. A cross‐sectional survey in Shanghai, China, shows more than 50% of health professionals need training on disaster management [25]. A study done in Ethiopia also showed that only 20.6% of the respondents were trained on disaster‐related topics [23]. According to a study, the Croatian majority (73.8%) of physicians have not participated in any disaster‐related educational activity [26].

The most common disaster reported by health professionals in this study was transport accidents, which were about 39.5%, followed by environmental pollution at 35.4%.

### 5.2 Factors Associated With Disaster Preparedness

The present study found that participants having midwifery and medical laboratory specialties were significantly associated with having adequate knowledge of emergency preparedness. About 9.7% of midwifery, 2.4% medical laboratory and nearly 7.8% of pharmacy specialty health professionals had adequate knowledge of emergency preparedness, which was still low. It is known that every health professional should have adequate knowledge to prepare themselves for early responding to the problem without costing more human lives.

Study participants with disaster volunteering were also significantly associated with adequate knowledge of emergency preparedness. Approximately 5.4% of participants with disaster volunteers had adequate knowledge of emergency preparedness. It is true that if health professionals volunteer for disaster management, they are meant ready for those emergency situations and take training regarding disaster management.

Having 1–5 years of working experience was also significantly associated with adequate knowledge of emergency preparedness. From this study, people having 1–5 years were 2.54 times more likely to have adequate knowledge of emergency preparedness. This may be due to that, as the health professional would learn from their previous experience of emergency management.

Participants greater than 30 years of age were also significantly associated with adequate knowledge of emergency preparedness. The findings of this study showed that respondents with an age group greater than 30 years of age were 3.69 times more likely to have adequate knowledge of emergency preparedness. This may be due to that this age group, health professionals may get training throughout their career and may face problems in their stay. Additionally, they may be highly energetic and there is even burnout in their career.

### 5.3 Strengths and Limitations of the Study

#### 5.3.1 Strength of the Study

- This study used standardized tools.
- Inclusion of a relatively large sample size and using an objective data collection method.

#### 5.3.2 Limitations of the Study

✓ This study had a few limitations, including its cross‐sectional design.
✓ There might be social desirability and recall bias.

## 6 Conclusion and Recommendations

This study finding shows that about 57.6% of health professionals working in North Shewa, Oromia, Ethiopia had adequate knowledge of emergency preparedness. There was a likelihood of frequent transport accident cases in the area thereby leading to death unless a lot of emphasis is put on awareness creation about emergency preparedness. Health professionals with a midwifery specialty, medical laboratory specialty, pharmacy specialty, disaster volunteer, 1–5 years of experience and greater than 30 years of age were significantly associated factors with adequate knowledge of emergency preparedness. Therefore, training regarding emergency preparedness should be given to all health professionals, including midwifery, medical laboratory and pharmacy, so as to increase their knowledge level. The local health department also recommended working on creating many disaster volunteer health professionals to solve the problem.

## Author Contributions

The ideal conception, study design, data acquisition, analysis, and interpretation were all contributed to by all the authors. All the authors have reviewed the manuscript and approved its publication in this journal.

## Funding

The authors received no specific funding for this work.

## Ethics Statement

Salale University Research and Ethical Review Committee approved the research proposal with a letter. Before data was collected, permission letters were obtained from hospital managers and the Woreda's health office, and each study participant gave their verbal agreement voluntarily. Above all, this study was entirely conducted as per the Declaration of Helsinki ethical principles for medical research on human subjects.

## Conflicts of Interest

The authors declare no conflicts of interest.

## Transparency Statement

The lead author Melese Wagaye Zergaw affirms that this manuscript is an honest, accurate, and transparent account of the study being reported; that no important aspects of the study have been omitted; and that any discrepancies from the study as planned (and, if relevant, registered) have been explained.

## Data Availability

The data that support the findings of this study are available from the corresponding author upon reasonable request.
